# Clinical and experimental studies of potentially pathogenic brain-directed autoantibodies: current knowledge and future directions

**DOI:** 10.1007/s00415-014-7600-8

**Published:** 2014-12-10

**Authors:** James Varley, Angela Vincent, Sarosh R. Irani

**Affiliations:** Nuffield Department of Clinical Neurosciences, John Radcliffe Hospital, West Wing, Level 6, Oxford, OX3 9DU UK

**Keywords:** Autoimmune encephalitis, Autoantibodies, Cell-surface, LGI1, CASPR2, NMDA-receptor

## Abstract

The field of neuronal surface-directed antibody-mediated diseases of the central nervous system has dramatically expanded in the last few years and now forms an important cluster of treatable neurological conditions. In this review, we focus on three areas. First, we review the demographics, clinical features and treatment responses of these conditions. Second, we consider their pathophysiology and compare autoantibody mechanisms and their effects to genetic or pharmacological disruptions of the target antigens. Third, we discuss areas of controversy within the field, propose possible resolutions, and explore new directions for neuronal surface antibody-mediated diseases.

## Introduction

Neuroimmunology is a rapidly developing field with increasing scope and relevance to multiple neurological presentations. Autoantibody-associated neurology has expanded since the discovery of pathogenic acetylcholine receptor autoantibodies in myasthenia gravis in the 1970s, and subsequently other neuromuscular and peripheral nerve targets.

The first antibodies associated with diseases of the central nervous system (CNS) were termed ‘onconeuronal’ antibodies due to their frequent cancer associations [1, 2]. These antibodies target intracellular proteins (such as Hu, Yo, Ma2, Ri, Tr and CV2/CRMP5), the antibody levels do not correlate with disease severity, and prognosis is poor despite tumour removal and immunotherapies. A cytotoxic T cell-mediated mechanism is thought to be central to their pathophysiology and the role of the antibodies is less clear.

These features contrast markedly with the neuronal surface-directed antibody (NSAb)-associated CNS disorders. The antibodies are much less frequently associated with tumours, and are directed against extracellular epitopes on surface antigens strongly expressed within the CNS, such as the *N*-methyl-d-aspartate receptor (NMDAR) [3] and leucine-rich glioma-inactivated 1 (LGI1) [4]. The discovery of these NSAbs has helped identify treatable neurological conditions, with retrospective evidence that earlier treatment improves patient outcomes [5]. Although research is rapidly evolving, the available data strongly support pathogenic roles for the NSAbs. The antibody targets can be divided empirically into three groups: excitatory neurotransmitter receptors, inhibitory neurotransmitter receptors, ion-channel subunits or cell adhesion molecules.

## NSAbs and their clinical features

### Antibodies directed against proteins involved in excitatory neurotransmission

#### NMDAR

Since their discovery in 2007 [3], NMDAR-antibodies now represent a more frequent cause of encephalitis than viruses in patients under the age of 30 [6]. This encephalitis shows a stereotyped evolution from a viral prodrome to a neuropsychiatric presentation, with psychosis, cognitive dysfunction and seizures, followed by a progression to a distinctive movement disorder, dysautonomia and coma [7].

**Fig. 1:**
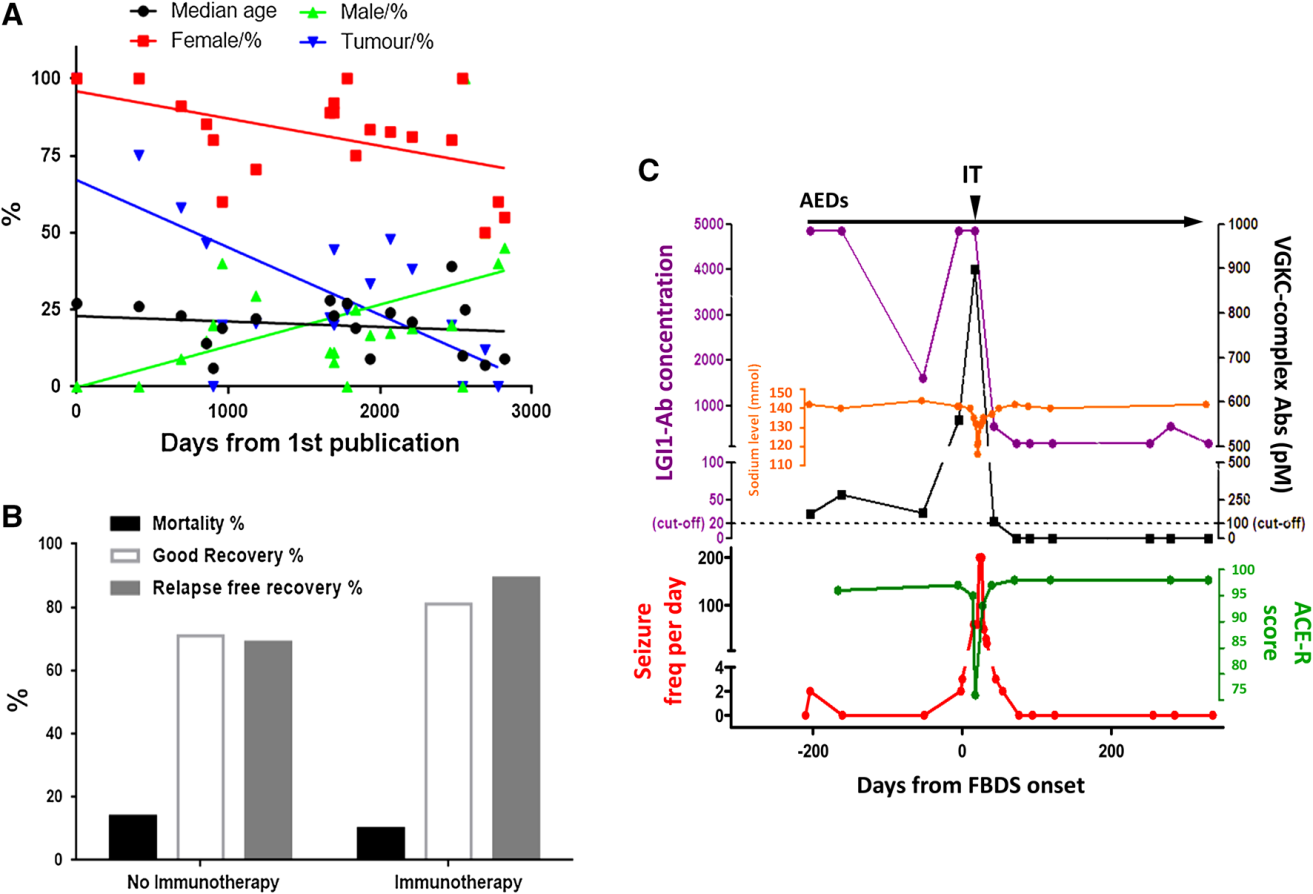
**a** Trends in NMDAR-antibody encephalitis. Demographics of published cases (series containing >3 patients) with NMDAR (*N*-methyl-d-aspartate receptor)-antibody encephalitis. Note the slightly decreasing median age (*black line*) and increasing male and falling female representation (*green* and *red*, respectively). Tumour frequencies (*blue line*) are falling, mainly due to the recent publications of many paediatric cases. Figure adapted from Irani et al. [31]. **b** The effect of immunotherapy on mortality, the percentage with a good recovery (modified rankin score 0–2) and relapse-free recovery at 24 months. Data derived from Titulaer et al. [5]. **c** Key features of a representative patient with faciobrachial dystonic seizures (*FBDS*). Note the increasing seizure frequency (*red line*), poor response to anti-epileptic drugs (*AEDs*), time of onset of cognitive impairment (quantified by fall in Addenbrooke’s cognitive examination-Revised score (*ACE-R*, *green line*)) and of hyponatraemia (*orange line*). IT results in dramatic improvement in all features. *LGI1* leucine-rich glioma-inactivated, *VGKC* voltage-gated potassium channel—complex antibody titres are shown in *purple* and *black*, respectively

Since its original description the spectrum has widened and this disease has been associated with fewer tumours (Fig. 1a), almost all ovarian teratomas [7, 8], increasing numbers of paediatric cases (with only a 6 % association with a tumour seen in those under 12 [5, 7]), and more male cases, particularly in younger and older age groups [5, 7, 9].

Mono- or oligo-symptomatic presentations in patients with NMDAR-antibodies have also been recognised with predominant seizures and psychosis [7, 8, 10, 11]. Other presentations seen in a small proportion of NMDAR-antibody-positive patients include longitudinally extensive transverse myelitis [12] and optic neuritis [13]. This overlap with demyelinating diseases may relate to the expression of NMDARs on oligodendrocytes. However, an overlap with neuropsychiatric lupus, where double-stranded DNA antibodies have been reported to cross-react with the NMDAR [14, 15], is yet to be confirmed using cell-based assay techniques (discussed below) [3, 7].

**Fig. 2:**
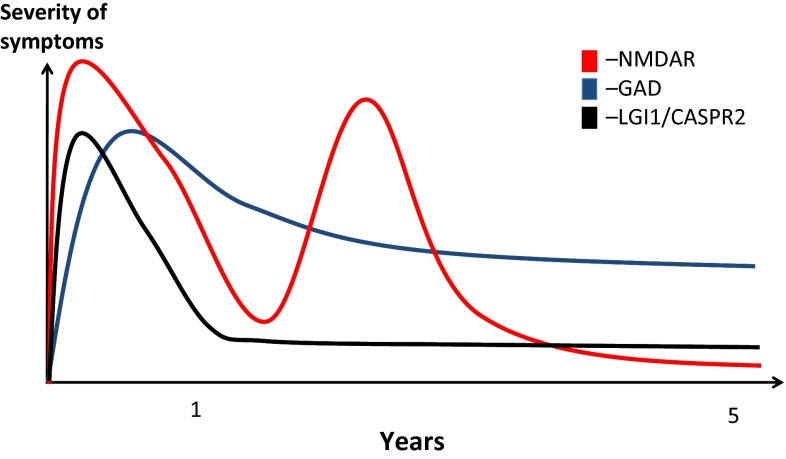
The contrasting probable natural histories of three antibody-related encephalitidies. Key things to note are the relapsing course *NMDAR* (*N*-methyl-d-aspartate receptor)-antibody encephalitis, often with a good long-term outcome. The *LGI1* (leucine-rich glioma-inactivated 1) or *CASPR2* (contactin-associated protein 2)-associated encephalitis has a tendency to be more monophasic often with residual memory and functional deficits. *GAD* (glutamic acid decarboxylase)-antibody-associated LE has an insidious *onset* and tends to adopt a more chronic course with ongoing seizures and memory deficits

NMDAR-antibody encephalitis has an approximately 13 % untreated mortality, as compared to 9 % with immunotherapy (Fig. 1b) [5]. One large study showed that 50 % of patients responded to first-line therapy [with corticosteroids, plasma exchange (PLEX) and/or intravenous immunoglobulin (IVIG)]. Of the remaining 50 %, second-line therapies (with cyclophosphamide and rituximab) offered a good outcome in 37.5 % compared to the 12.5 % that did not receive such therapies. Immunotherapy administration was also associated with lower relapse rate, often seen in the natural history of this disease (Figs. 1b, 2) [5]. While it yet may transpire that immunotherapy has little effect on the long-term outcomes of the disease survivors, importantly it appears to hasten recovery at 2 years and reduce mortality.

### α-Amino-3-hydroxy-5-methyl-4-isoxazolepropionic acid (AMPA-type (glutamate receptor)) receptor

Antibodies to the GluR1 and GluR2 AMPA-receptor subunits often associate with a rare limbic encephalitis (LE) in older females, typically with tumours of the thymus, breast and lung [16]. The syndrome of LE produces amnesia, disorientation and seizures and is also seen in patients with GAD, LGI1 and GABA_B_R-antibodies, as discussed later. Some patients show a good response to immunotherapy [16]. All examined tumours expressed at least one of the antigens, and a predominance of one subunit in the tumour mirrored the antibody preference seen in the same patient [16].

AMPA receptors are usually tetramers of GluR subunits 1–4. GluR1/2 and GluR2/3 subunits are mostly post-synaptic and are expressed at especially high concentrations in limbic brain regions [17]. AMPAR-antibody-associated phenotypes have spread to include two patients with an acute psychosis-like illness [18] and antibodies to GluR2/3 receptors were found in two patients with Rasmussen’s encephalitis [Nibber et al. in preparation].

### Antibodies to proteins involved in inhibitory neurotransmission

#### *GAD*

Glutamic acid decarboxylase (GAD) is a widely expressed intracellular enzyme which catalyses the synthesis of gamma aminobutyric acid (GABA), the major inhibitory CNS neurotransmitter. Antibodies to GAD are seen in type 1 diabetes mellitus, and usually at much higher titres in LE, cerebellar ataxia, epilepsy and the stiff person syndrome (SPS) spectrum [19].

SPS is characterised by rigidity, stimulus-induced spasms, anxiety, and more rarely, oculomotor and autonomic disturbances [19, 20]. By contrast, GAD-antibody-associated LE is predominantly a disease of young women and usually presents with AED-refractory epilepsy and amnesia, but without rigidity or spasms. The clinical features and GAD-antibody levels are often immunotherapy resistant, and the disease shows a chronic course (Fig. 2) [21]. However, serum and CSF IgGs from patients with GAD-antibodies do reproduce some of the clinical features of SPS in rodents [22]. The antibodies may access antigen upon its cell-surface exposure during exocytosis or programmed cell death [23, 24] or perhaps co-exist with pathogenic NSAbs. Indeed, antibodies to the AMPA, glycine, GABA_B_ and GABA_A_ receptors, in addition to novel/undefined NSAbs, have all been observed in patients with GAD-antibody-related neurology [25–31].

#### Glycine receptor

Progressive encephalomyelitis with rigidity and myoclonus (PERM) is at one end of the SPS spectrum with the poorest prognosis, and usually these patients have no GAD-antibodies [30]. In 2008, a patient with PERM without GAD-antibodies was found to have antibodies directed against the glycine-receptor (GlyR) alpha1 subunit [32]. Subsequently, GlyR-antibodies have been reported in patients with classical and variant SPS, brainstem encephalitis, a few with LE, many with PERM, and occasionally in patients with demyelinating disease. There is a good response to immunotherapy (median modified Rankin Scale scores fall from of 5 to 1) [27, 33, 34]. Tumour associations are infrequent but thymoma and lymphoma have been reported [27]. The GlyR is expressed in the upper and lower brainstem, diencephalon and the colliculi as well as the dorsal and ventral horns of the spinal cord: these localisations correlate well with the observed clinical features [27].

#### *GABA*_*B*_ receptor

GABA_B_-antibodies, predominantly reacting with the GABA_B1_ subunit, have been associated with a form of LE, usually of later life, with prominent seizures [25, 26]. More recently the phenotype has expanded to include presentations with cerebellar ataxia, status epilepticus, and opsoclonus myoclonus, often in patients with cognitive impairment [25, 35]. There is a close association with small-cell lung cancers (SCLC) [25, 26, 36], which express the GABA_B_R [25]. Mortality is high, especially in tumour-related cases, but 80 % of patients initially respond to immunotherapy, plus tumour removal where relevant [25].

#### GABA_A_ receptor

Antibodies to the GABA α1/β3 subunits have recently been described in a small number of patients. When detected at high serum titres (>1:160) and in the CSF, these were associated with LE, status epilepticus or epilepsia partialis continua [28]. Patients have unusual cortical and subcortical imaging hyperintensities, a variable response to immunotherapy, and high mortality due to status epilepticus. Twelve patients with other neurological diseases had lower titre serum GABA_A_-antibodies, not detected in the CSF, with a broader spectrum of diseases including LE, SPS, and opsoclonus myoclonus [28]. Autoantibodies against the α1 and/or γ2 subunits were found in patients with seizures (47 %), memory impairment (47 %) and hallucinations (33 %); one had non-Hodgkin’s lymphoma (Pettingill et al. in press). In that study, however, many patients were not considered to have immune-mediated diseases, and immunotherapies were not used in most. Nevertheless, the antibodies internalised the GABA_A_R subunits in vitro, consistent with their pathogenic potential [37] (Pettingill et al. in press).

### Antibodies directed against ion-channel-associated proteins and cell adhesion proteins

#### Voltage-gated potassium channel (VGKC) complex

**Fig. 3:**
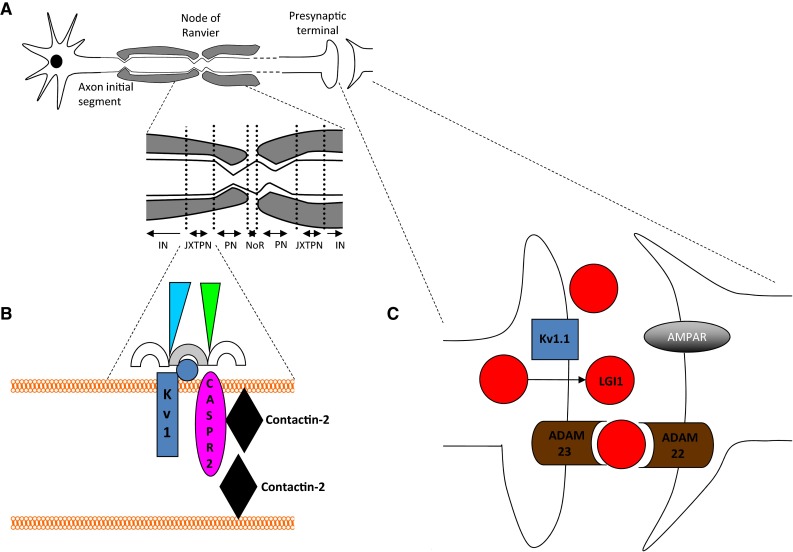
Illustration of the VGKC-complexes: the association of *Kv1*s and *CASPR2* (contactin-associated protein), *LGI1* (leucine-rich glioma-inactivated and other components of the complexes. **a** Neuronal subcellular domains including the axon initial segment, presynaptic terminal, node of Ranvier (*NoR*), paranode (*PN*), juxtaparanode (*JXTPN*) and internode (*IN*). **b** Juxtaparanode: Kv1 channels (*blue*, alpha subunits = *rectangle*, beta subunit = *circle*), CASPR2 (*pink oval*), contactin-2 (*black diamond*), MAGUKs (membrane-associated guanylate-kinases) (*semicircles*), protein 4.1B/spectrins/ankyrins (*green*/*blue triangles*). **c** Synaptic Kv1 organisation. Kv1 s (*blue*, such as Kv1.1), LGI1 (*red*) and α-amino-3-hydroxy-5-methyl-4-isoxazolepropionic acid receptors (*AMPAR*) and ADAM22/23 (a disintegrin and metalloproteinase 22/23) (*brown*) anchored at post-synaptic membranes

Antibodies to the VGKC-complex were originally described in patients with peripheral nerve hyperexcitability (PNH) syndromes [38]. Since 2001, these antibodies have been recognised in patients with CNS features including Morvan’s syndrome (MoS) [39, 40], LE [4, 41, 42], faciobrachial dystonic seizures (FBDS) [43–45], a minority of patients with cryptogenic epilepsies [46], neuropathic pain syndromes [47] and some cerebellar ataxias [48]. VGKC-complex antibodies are detected by immunoprecipitation of iodinated alpha-dendrotoxin (α-DTX)-labelled VGKCs from digitonin-solubilised mammalian brain homogenates. α-DTX is known to bind with high affinity to the VGKC subunits Kv1.1, 1.2 and 1.6. Based on this, Kv1.1, 1.2 and 1.6 were considered the likely target epitopes [49]. However, only a minority of IgGs bind the Kv1 subunits themselves [4]. A much larger proportion bind to target cell-surface domains of proteins which are tightly associated with Kv1 subunits; most commonly LGI1 and contactin-associated protein-2 (CASPR2) [4, 39, 50] (Fig. 3). A smaller proportion were found to target contactin-2, which have been reported in association with LGI1- and CASPR2-antibodies [4].

#### LGI1

LGI1 is a secreted protein that interacts in situ with Kv1.1, Kv1.2 and AMPARs. LGI1 forms a trans-synaptic protein complex with presynaptic ADAM23 (a disintegrin and metalloproteinase 23) and post-synaptic ADAM22 [51]. It is expressed throughout the brain, especially in the hippocampus and neocortex [4].

LGI1-antibodies are often found in LE [4, 52]. As the descriptions of LGI1-antibody-positive cohorts have grown, it has become increasingly clear that this is only rarely paraneoplastic, has an equal sex distribution, responds well to immunotherapy, and has a low overall mortality [4, 45, 50, 53] (see Table 1). Although there are descriptions of untreated partial recovery over around 2 years [54, 55], larger cohorts suggest that early immunotherapy offers the best short-term outcomes [42, 56], and recent data indicate that the addition of PLEX and/or IVIG to corticosteroids may not alter 4-year outcomes [31].

**Table 1:** The key clinical aspects of the major neuronal surface-targeted antibodies based on current available evidence from published patient series

	NMDAR	LGI1	CASPR2	GAD_1_	GlyR	GABA_B_
Median age of onset	21 (2–76) [5, 7, 8]	68 (28–92) [45, 50]	57 (19–80) [39]	23 (17–66) [21]	50 (1–75) [27]	62 (24–75) [25, 36]
Gender	F > M (70–90 %) [5, 7, 8]	Equal [4]	M > F ~90 % [4, 39, 107]	F > M ~80 % [21]	Equal [27]	Equal [25]
Clinical features	Frequent stereotyped progression from early cognitive dysfunction, psychiatric features and seizures, to later movement disorder, autonomic failure and reduction in consciousness [8]	FBDS LE [50] Often present in Morvan’s syndrome (coexisting with CASPR2-antibodies). Isolated epilepsy in some [59]	Morvan’s syndrome Neuropsychiatric features, insomnia, autonomic failure and neuromyotonia (often with LGI1 antibodies) [39, 107] Around 10 % have a cerebellitis [48] Around 10 % of VGKC-complex antibody-positive LE [4, 53] Isolated epilepsy noted (Irani unpublished)	Stiff person syndrome > cerebellar ataxia > LE > isolated epilepsy [19]	Stiff Person syndrome spectrum of diseases: PERM > vSPS > cSPS [27] Rarely LE [27]	LE, predominantly with seizures as presenting symptom [25, 36] Rarely cerebellar ataxia, status epilepticus or opsoclonus myoclons—usually progresses to LE [108]
Investigation findings	MRI normal in ~50–75 % [5, 7, 8] LP abnormal in 80–90 % [5, 7, 8] EEG abnormal in 80–90 % [5, 7, 8]	MRI abnormal in >60–80 % [4, 45, 50] LP Normal [45] Hyponatraemia in 60-80 % [4, 45, 50] EEG abnormal in 80 %	MRI normal in >60–90 % [4, 39] LP abnormal in 50 % [39] Hyponatraemia in 10–25 % [4, 39, 107] EEG abnormal in ~60 % if central involvement [107]	MRI abnormal in 100 % (used as selection criteria in this study) [21] LP abnormal in 22 % [21]	MRI Head abnormal in 30 %, spine in 20 % [27] LP abnormal in 60 % [27] EMG abnormal 60 % [27] EEG abnormal in 70 % [27]	MRI abnormal in ~75 % [25] LP abnormal in 60 % [25] EEG abnormal in ~90 % [25]
Tumour association	Ovarian teratoma in 30 [7] –50 % [5, 8] 6 % in <12-year olds [5]	<10 % (various tumours observed) [4, 50]	Thymoma ~40 % of Morvan’s [39] Tumour <10 % in other presentations [107]	None [21]	Thymoma <10 % [27]	50 % (lung cancer, predominantly small-cell lung cancer) [25, 108]
Immunotherapy efficacy	First-line IT leads to good outcome in 81 %, no need for ITU stay and early IT associated with better outcome [5, 8]	Steroids and PLEX reduce cognitive impairment and decrease disability [4, 45, 52]	IT reduces disability, less response if thymoma [4, 39, 107]	Poor efficacy—0 % seizure freedom [21]	90 % shows good response [27]	80 % response rate to IT ± tumour resection (if treated) [25, 108]
Prognosis (inc relapses)	6 % mortality (12 % if untreated) [5] 12–15 % relapse (median time to relapse 18 months [1–84 months]) [5, 8]	2 % mortality [4]; low rates of relapse unless treatment is withdrawn early	20–31 % mortality, (highest risk if thymoma) 7 % relapse rate [4, 39]	Low mortality, due to poor response to Rx, relapse not able to be defined [21]	10 % mortality (highest risk if thymoma) [27] 11 % relapse rate [27]	30 % mortality (predominantly tumour or chemotherapy related) [25, 26]

	AMPAR	AQP4	MOG	GABA_A__2_	DPPX	IgLON5
Median age of onset	60 (38–87) [16]	37 (4–78) [106]	37.5 (3–70) [106]	22 (3–63) [28]	53 (13–76) [68, 69]	59 (52–76) [70]
Gender	F > M ~90 % [16]	F > M (90 %) [106]	M > F (60 %) [106]	M > F (80 %) [28]	Equal [68, 69]	Equal [70]
Clinical features	LE	NMOSD	NMOSD—more likely to have optic neuritis, especially bilaterally [106]	Status epilepticus or epilepsia pars continua at high titres [28] LE [37]	LE with tremor, myoclonus, brainstem dysfunction and dysautonomia with 50–75 % having diarrhoea of unknown cause [68, 69]	REM and non-REM sleep disorder, sleep breathing disorder, movement disorder. Occasional rapid brainstem degeneration with dysautonomia, central hypoventilation, dysphagia and dysarthria [70]
Investigation findings	MRI abnormal in 80 % [16] LP abnormal in 90 % [16] EEG abnormal is 60 % [16]	MRI abnormal 60 % [106] LP oligoclonal bands in 20 % [106]	MRI abnormal 40 % [106] LP oligoclonal bands in 0 %, more likely to have higher cell count [106]	MRI abnormal in 100 % [28] LP abnormal in 84 % [28] EEG abnormal 100 % [28]	MRI abnormal in 66 % [68] EEG abnormal in 100 % [68] LP abnormal in 100 % [68]	Videopolysomnography—OSA, stridor and abnormal sleep architecture Neuropathology—neuronal loss and atypical brainstem tau deposition [70]
Tumour association	Lung, breast, thymoma ~70 % [16]	Rare, various infections can trigger relapse	None	16 % treated for Hodgkin’s lymphoma 10 months previously [28]	None	None
Immunotherapy efficacy	90 % response rate to IT ± oncological therapy. [16]	80 % response rate to IT, reduction in relapse rate with use of Rituximab [109–111]	Immunoresponsive [106]	50 % recovery with IT [28]	100 % gradual response to IT [68]	No response to immunotherapy [70]
Prognosis (inc relapses)	30 % mortality (higher if tumour and no treatment) 50 % relapse rate (median time to relapse 16 months) [16]	70 % have relapsing course [112]	50 % relapse (4.5 year median follow up) [106]	Mortality 33 % [28] 16 % relapse [28]	No mortality 100 % relapse rate off IT [68]	Progressive

Above data calculated by taking into account a composite figure from the largest available data series on each antibody. 1. Demographics and clinical information for anti-GAD centres on GAD LE. 2. High titre patients included only for clinical information sections

*NMDAR N*-methyl-d-aspartate receptor, *LGI1* leucine-rich glioma-inactivated 1, *CASPR2* contactin-associated protein 2, *GAD* glutamic acid decarboxylase, *GlyR* glycine receptor, *GABAB* γ-aminobutyric acid B, *AMPAR* a-amino-3-hydroxy-5-methylisoxazole-4-propionic acid receptor, *AQP4* aquaporin-4, *MOG* myelin oligodendrocyte glycoprotein, *GABAa* γ-aminobutyric acid A, *DPPX* dipeptidyl-peptidase-like protein-6, *LE* limbic encephalitis, *FBDS* faciobrachial dystonic seizure, *SPS* stiff person syndrome, *vSPS* variant stiff person syndrome, *cSPS* classical stiff person syndrome, *NMOSD* neuromyelitis optica spectrum disorders, *REM* rapid eye movement, *OSA* obstructive sleep apnoea, *LP* lumbar puncture, *EEG* electroencephalogram, *EMG* electromyelogram, *PLEX* plasma exchange, *IT* immunotherapy

Several studies have described LGI1-antibodies in patients with isolated seizure syndromes of multiple semiologies, which are often immunotherapy-responsive [57–59]. A recent clinical observation has been the association of a highly distinctive seizure semiology—termed faciobrachial dystonic sseizures (FBDS)—in patients with LGI1-antibodies. These stereotyped events, characterised by their high frequency (median 50/day), short duration (usually <3 s) and their predilection for the hemiface and ipsilateral arm, are often refractory to anti-epileptic drugs but preferentially respond to the addition of immunotherapies (see Fig. 1c) [43–45, 60, 61]. Importantly, the onset of FBDS often precedes the onset of the cognitive impairment seen in patients with LE and one small prospective study has suggested that cognitive impairment may be avoided with early treatment of FBDS [43–45, 60]. In addition, ictal bradycardia and piloerection may be seizure semiologies enriched in patients with LGI1-antibodies [62, 63].

#### CASPR2

CASPR2 is a transmembrane protein localised to the juxtaparanode of myelinated axons. The extracellular domain of CASPR2 interacts with contactin-2 in both *cis* and *trans* (Fig. 3b), and in association with other proteins is responsible for concentrating Kv1.1 and Kv1.2 channels at the juxtaparanode [64]. Therefore, CASPR2 has cell adhesion and Kv1-partner functions. Patients with LE, PNH and subacute cerebellitis [48] have CASPR2-antibodies in around 10, 30 and 10 % of cases, respectively. However, CASPR2-antibodies are most consistently associated with MoS, in which about 50 % of patients also have LGI1-antibodies [39]. This combination may generate both the CNS and PNS features of MoS. MoS occurs almost exclusively in males, and interestingly, the prostate is one of the few non-neuronal sites of CASPR2 expression and CASPR2-antibody-associated MoS has been described post-scrotal hydrocele drainage [65]. Another potential immunisation mechanism is via the associated thymomas present in around 50 % of MoS and especially in patients with CASPR2-antibodies [39].

#### Other VGKC-complex proteins

Most VGKC-complex antibody-positive patients with higher VGKC-complex titres (>400 pM), have LGI1- or CASPR2-antibodies. Much more commonly at lower levels (100–400 pM), the targets of the antibodies are not yet known [4, 53]. Serum and CSFs from many of these patients do not show binding to the surface of live hippocampal neurons (Vincent, unpublished), suggesting that they may target intracellular VGKC-complex epitopes. While these may not be pathogenic, the antibodies may still be predictive of a neuroinflammatory syndrome and a response to immunotherapy, or an inflammatory component to a neurodegenerative disease [66, 67] (Hacohen et al. submitted).

#### Dipeptidyl-peptidase-like protein-6 (DPPX)

A subacute LE associated with tremor, myoclonus and diarrhoea was described in association with antibodies to DPPX, a cell-surface protein associated with the Kv4.2 potassium channel [68]. A more recent study has highlighted the brainstem focus of this condition and the multiorgan dysautonomia with bladder and cardiac involvement [69]. The condition is usually severe, with a gradual response to immunotherapy and relapses without immunotherapy [68].

#### IgLON5

Not all NSAbs are pathogenic. Antibodies to IgLON5, a neuronal cell adhesion protein involved in synapse formation, were described in patients with a progressive complex neurodegenerative sleep disorder with disordered breathing [70]. The most striking aspect of these patients was their lack of response to immunotherapy and atypical brainstem tau deposition [70]. The association of an NSAb with a neurodegenerative disease adds to evidence from CJD studies [71, 72] that NSAbs may not always play a primary role but can be secondary to neuronal damage with possible implications for disease progression or disease biomarkers.

Antibodies associated with glial damage, specifically to aquaporin-4 (AQP4) and myelin oligodendrocyte glycoprotein (MOG), are summarised in Table 1.

## Pathogenic considerations

**Fig. 4:**
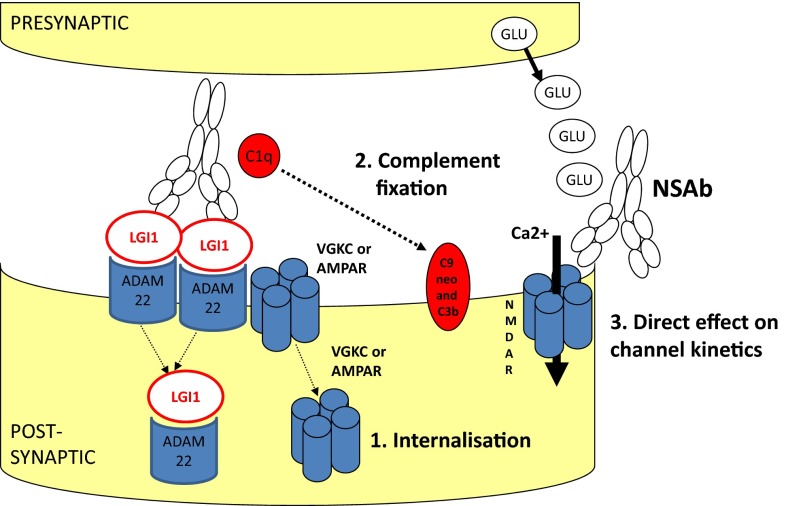
Potential pathogenic mechanisms of neuronal surface-directed antibodies (*NSAbs*). **a** Internalisation of receptors has been demonstrated in vitro using *NMDAR* (*N*-methyl-d-aspartate receptor), *AMPAR* (a-amino-3-hydroxy-5-methylisoxazole-4-propionic acid receptor) and GABA_A_R (γ-aminobutyric acid A receptor)-antibodies. Here the LGI1–ADAM22 interaction is shown as a possible unit for co-internalisation. **b** Antibody-mediated complement fixation and complement-mediated membrane receptor disruption as seen with antibodies against AQP4 (aquaporin-4). **c** Direct alteration of ion-channel molecular function is an alternative mechanism

Exactly how these antibodies lead to the observed pathology is an area of active research (Fig. 4). For instance, many but not all NSAbs induce receptor internalisation in vitro resulting in receptor loss, as observed in myasthenia gravis. This applies to NMDAR [8], AMPAR [16], GABA_A_R [37] and GlyR [27] antibodies and can be demonstrated in cell cultures. Other NSAbs may mediate a direct effect on channel kinetics. LGI1- and DPPX-antibodies may indirectly induce channel modulation: for example, LGI1-antibodies appear to reduce Kv1 channel function [73] and to decrease AMPAR expression in vitro [74]. It is likely that the antibodies binding to LGI1 disrupt the trans-synaptic bridge between the pre- and post-synaptic membranes and this may affect the function of both VGKCs and AMPARs. In addition, however, antibodies of the IgG1 and IgG3 subclasses have the ability to fix complement. In biopsy studies, this has been shown to occur with AQP4- (and less so LGI1-) antibodies but not with NMDAR-antibodies [75, 76]. Mechanisms that appear to prevent complement fixation by the IgG1-subclass NMDAR-antibodies should be explored in future studies.

To further explore NSAb-pathogenicity, below we highlight features of genetic or pharmacological situations in which the target antigen is relatively specifically disrupted and compare this to the corresponding antibody-mediated process (Table 2).

**Table 2:** Key characteristics of drugs and mutations affecting key antigens in antibody-mediated encephalitis

	LGI1-animal mutant	LGI1-human mutant	NMDAR-animal mutant	NR1 human polymorphism	NMDAR—drugs, e.g. ketamine/phencyclidine	CASPR2-animal mutant	CASPR2-human mutant	GABA_B1_R drugs—baclofen	GABA_B1_R animal mutant	GABA_B1_R human polymorphism
Cognitive	Not specified	Unaffected interictally [113]	Impaired memory [78]	Associated with nonsyndromic intellectual disability [114]	Impaired concentration, semantic and episodic memory [115, 116]	Behavioural inflexibility, communication and social ability impairment [91]	Intellectual disability, inattention [93]	Impaired visual learning [79]	Heterozygotes have impaired pre-pulse inhibition, learning and hyperalgesia [80, 117]	N/A
Psychiatric	Inactivity, slow walking [84]	Unaffected interictally [113]	Anhedonia, anxiety, psychomotor agitation [78]	Associated with alcoholism [118], schizophrenia [119] and sensitivity to drug induced psychosis [120]	Hyperactivity, severe agitation, psychosis [115]	Hyperactivity and repetitive behaviour [91]	Hyperactivity, aggression, autism [93]	No effect on anxiety [79]	N/A	Some evidence suggestive of link to schizophrenia and obsessive–compulsive disorder [82, 83]
Seizures and semiology	Homozygous null mice myoclonic seizures [84]	Brief aphasic seizures with auditory hallucinations, nocturnal tonic–clonic seizures [87]	None noted	de novo mutation found in childhood epileptic encephalopathy [121]	Seizures reported rarely [122]	Seizures reported after 6 months of age [91]	Frequent, intractable predominantly focal seizures often with secondary generalisation [93]	Seizures [123]	Null mice develop generalised tonic–clonic seizures [117], Heterozygotes no seizures [117]	Association to temporal lobe epilepsy [81]
Other features	Null mice die at 12–18 days [84]	MRI unremarkable [113] and benign outcome [86]	NMDA null mice die of severe hypoventilation [77]	N/A	Impaired conscious level, hypoventilation, autonomic dysfunction [124] and movement disorder [125–127]	Normal nerve conduction studies with no evidence of hyperexcitability [90]	N/A	Impaired locomotion [79]	Null mice have reduced body weight, impaired locomotion [80, 117]	N/A
Neuronal abnormalities	Enhanced excitatory synaptic transmission with excess glutamate release [84]. Mice expressing mutant LGI1 mice have impaired synaptic pruning and functional maturation [85]	Not specified	Disinhibition of cortical excitatory neurons and reduced neuronal synchrony [78]	N/A	Impaired synaptic plasticity [115]	Loss of K+ channel clustering at juxtaparanodes [90], neuronal migration defect, loss of interneurons and asynchronous neural networks [91]	Widespread cortical dysplasia [93]	N/A	Null mice have histologically normal brains [117]	N/A

### **Mutations, drugs and antibodies which target the NR1 subunit**

NR1 homozygous null mice die 8 h after birth and hypoventilate, similar to patients with NMDAR-antibody encephalitis [77]. Mice with a 50 % NR1 genetic knockout exhibit both psychiatric and cognitive signs, similar to those seen in NMDAR-antibody encephalitis but without a movement disorder or seizures [78]. As described in Table 2, polymorphisms and de novo mutations in the human NR1 subunit gene (GRIN1), and NMDAR-antagonists such as ketamine and phencyclidine, recapitulate many aspects of NMDAR-antibody encephalitis. However, the ‘full’ syndrome appears to be unique to autoantibodies targeting the NR1 subunit.

### GABA_B_ receptor mutations and medications

Pathophysiologically, GABA_B_R-antibody LE shows predominant seizures. This concurs with observations from murine genetic and pharmacological studies of GABA_B_R downregulation [79, 80], but by contrast to most other antibodies GABA_B_R-antibodies do not appear to internalise their target antigen. There are no documented GABA_B1_R human mutants, but GABA_B1_R polymorphisms have been associated with temporal lobe epilepsy [81], schizophrenia [82] and obsessive–compulsive disorder [83].

### LGI1 mutations

Leucine-rich glioma-inactivated 1 homozygous null mice develop myoclonic seizures at days 12–18 of life, dying soon after [84]. Electrophysiological studies in both mutant LGI1 and LGI1 null mice demonstrate increased synaptic excitation [84, 85], thought to be mediated by increased glutamate efflux [84]. LGI1 mutations in humans cause autosomal dominant lateral temporal lobe epilepsy (ADLTE), with ictal auditory hallucinations [86]. Some patients have generalised tonic–clonic seizures, sensory aphasic seizures, and a few kindreds have ictal psychic phenomena [87].

Contrasts are stark between LGI1 human mutants and the corresponding antibody-mediated syndromes. In patients with LGI1 mutations, despite focal temporal lobe seizures, there are no cognitive or psychiatric manifestations, seizure semiology is predominantly auditory, and MRIs are normal. The opposites are true of patients with FBDS- and LGI1-antibodies. These differences may be accounted for by antibody access to only specific brain regions, the effect of complement-mediated neuronal damage, or genetic compensation in LGI1 mutants which may not occur in a rapid onset antibody-mediated disorder. Perhaps some effects of LGI1 mutations are via expression or function of VGKCs. Indeed, humans with Kv1.1 mutations have neuromyotonia and an increased rate of seizures [88, 89].

### CASPR2 mutants

Contactin-associated protein-2 null mice were originally thought to have normal behaviour and neuronal growth despite loss of K+ channel juxtaparanode clustering [90]. More recent analyses have shown that they demonstrate hyperlocomotion, repetitive and inflexible behaviours, impaired socialising and seizures after 6 months of age [91].

The findings are reminiscent of those seen in autistic spectrum disorders, and mutations/polymorphisms in the CASPR2-encoding gene, CNTNAP2, have been linked to autism [92]. Interestingly, a recessive non-coding mutation for CNTNAP2 causes cortical dysplasia focal epilepsy syndrome (CDFE), characterised by seizures, intellectual disability, hyperactivity, and in two-thirds of cases, autism [93]. Mutations in CNTNAP2 have also been linked to schizophrenia, psychosis and other forms of epilepsy [94]. Therefore, there are clear similarities between patients with CASPR2-antibodies and mutations in CASPR2.

## Controversies, possible resolutions and new directions

### Antibody levels and assay methodologies

Early in these illnesses, the levels of serum autoantibodies are almost always higher than CSF autoantibodies. This seems intuitive in patients with a peripheral tumour, such as an ovarian teratoma, and also given patients positive clinical responses after plasma exchange. Therefore, it seems likely that reports of autoantibody detection in the CSF, but not serum, are due to differences in assay methodologies. One consideration is that the presence of intrathecal autoantibody synthesis, particularly seen with NMDAR-antibodies, and the constitutively low total IgG levels in CSF makes CSF easier to use than serum in diagnostic assays. As differences between antibody-detection methods have been discussed in detail elsewhere [31, 95, 96], here we summarise the main areas of controversy.

Autoantibodies with pathogenic potential recognise the extracellular domains of native membrane proteins. They are very rarely detected in denaturing western blots. However, assays utilised in the field do not always exclusively detect these autoantibodies. For example, the use of fixed tissue (where native epitopes may be destroyed) and the use of permeabilised antigen-transfected cells to detect antibodies may permit non-pathogenic autoantibody binding [8, 96]. Despite this possibility, the concurrent use of live hippocampal neurons [4, 8] and techniques of antibody absorption exclusively against the extracellular domain [4, 97] allay this concern [31]. In conclusion, differences between current assays suggest that both CSF and serum should be sent to laboratories whenever possible, and future cross-laboratory comparative assays should help understand the differences described above.

### NSAbs in other neurological diseases and the healthy population

In a recent study by Dahm et al., sera from over 4,000 healthy and disease controls with varied neuropsychiatric presentations (including schizophrenia, ALS, Parkinson’s and stroke) were screened for a panel of NSAbs and intracellular-targeted antibodies. ~11 % of the combined cohort was found to be positive for IgM- (6 %), IgA- (5 %) and IgG (1 %)-NMDAR-antibodies at titres from 1:10 to 1:1,000 with equal proportions in disease and healthy controls. Other frequently detected antibodies were amphiphysin (2.0 %), CASPR2 (0.9 %), MOG (0.8 %), GAD65 (0.5 %), Ma2 (0.5 %), Yo (0.4 %) and Ma1 (0.4 %), also with similar frequencies in disease and healthy controls [98]. The use of a permeabilised cell-based assay without CSF testing may account for the lowered specificity. However, as these antibodies appear to be present in healthy controls, this study suggests that clinical syndrome classification remains key to defining disease-relevant autoantibodies with pathogenic potential. Indeed, the lack of gold standards for disease, independent of antibody positivity, for research purposes is a problem for future studies.

### IgA and IgM autoantibodies

Antibodies of the IgG class associate with all of the NSAb-mediated diseases discussed thus far. There have, however, been reports of IgA- and IgM-NMDAR-antibodies associated with slow cognitive impairment [99], psychosis and bipolar disorder [100]. These IgM-NMDAR-antibodies caused a reduction in cell survival and NR1 expression in cultured rodent neurons [100], suggesting pathogenic potential. One study suggested that 31 % of patients with IgG-NMDAR-antibodies also had IgA-NMDAR-antibodies [99].

### Autoantibody triggers: tumours, infections and neurodegeneration

As tumours often express the antigen of interest in paraneoplastic NSAb-associated conditions, they are the likely sites of antigen presentation. In addition, other paradigms for breaking immune tolerance have arisen.

After herpes simplex virus encephalitis (HSVE), children often suffer relapses which have recently been associated with NMDAR-antibodies [101, 102]. Relapses occurred a few weeks to months after the HSVE, phenotypically closely resemble classical NMDAR-antibody encephalitis, and appear to be immunotherapy-responsive. The NMDAR-antibodies were found alongside other novel NSAbs and may represent systemic immunisation after neuronal damage. Indeed in adults, generation of VGKC-complex, glycine receptor and NMDAR-antibodies has been observed in a small proportion (<5 %) of patients with rapid neurodegeneration as seen in CJD [72, 103]. A more elegant example of this is the observation that VGKC-complex antibodies (amongst others) are generated in abattoir workers after exposure to aerosolised porcine neural tissue [104]. Furthermore, experimental rodents exposed to inhaled brain tissue aerosol developed a similar clinical and serological profile to their human counterparts [105]. It is likely that these antibodies are secondary.

In summary, multiple triggers appear able to generate serum autoantibodies with pathogenic potential. Factors governing the antibody-pathogenicity may include their access to the brain/CSF compartments, concentrations, the duration of antibody production, and intrinsic individual patient thresholds.

## Conclusions

Neuroimmunology has moved on from its position 15 years ago where it principally reflected research into multiple sclerosis and the pace of change and growth seems set to continue. There are increasing numbers of NSAbs associated with defined conditions. With the majority of discoveries being recent, much work will be needed to hone these phenotypes, their pathophysiological basis, optimal treatments, prognosis and longer term management. There are still methodological issues to settle, such as the best way to test for NSAbs, the independent gold standards for diagnosis of each condition and the relevance of low antibody levels in patients with non-classical syndromes. There are still antibodies to be found, for example in the VGKC-antibody-positive patients without LGI1- and CASPR2-antibodies. The underlying immunological mechanisms remain only partly characterised and much work will address this in coming years.

In the meantime, work must go on by the bedside, with clinicians recognising signs which suggest an underlying autoimmune condition and initiating the testing to detect an antibody, novel or otherwise. Only with such collaboration and high-quality clinical work can progress continue to be made in the laboratory to reduce the impact of these often devastating diseases.
